# Quercetin Enhances the Antibacterial Activity of Polymyxin E Against MCR-1-Positive Bacteria by Inhibiting the Biological Functions of the Cell Membrane

**DOI:** 10.3390/ani15233491

**Published:** 2025-12-03

**Authors:** Yongjie Zhang, Liyang Guo, Shun Wang, Jie Zhang, Xinlei Ren, Rui Li, Jichang Li, Chunli Chen

**Affiliations:** 1 College of Veterinary Medicine, Northeast Agricultural University, Harbin 150030, China; 2 Heilongjiang Key Laboratory for Animal Disease Control and Pharmaceutical Development, Harbin 150030, China

**Keywords:** combined medication, MCR-1, *Escherichia coli*, quercetin, antibiotic resistance

## Abstract

In animal husbandry and livestock farming, with the spread of the plasmid-mediated MCR-1 gene, polymyxin E, as the last line of defense against drug-resistant Gram-negative bacteria, is facing severe challenges. This study investigated the in vitro and in vivo synergistic effects and mechanisms of QUE combined with polymyxin E against MCR-1-positive chicken *E. coli* JD37. In vitro experiments showed that QUE could restore the sensitivity of *E. coli* JD37 to polymyxin E (FIC = 0.34375) and enhance the bactericidal effect of polymyxin E by increasing cell membrane permeability, fluidity, and membrane potential, downregulating the expression of the AcrAB-TolC efflux pump and LPS-related genes. Molecular docking further identified the key residues for QUE binding to the MCR-1 protein. The in vivo chick infection model confirmed that combination therapy increased survival rates, reduced bacterial load in tissues, alleviated pathological damage, and decreased levels of intestinal inflammatory factors. Our results demonstrate the synergistic bactericidal effect of the QUE-polymyxin E combination against MCR-1-positive *E. coli* and elucidate its underlying mechanism.

## Simple Summary

Polymyxin E is widely considered the last resort for treating multidrug-resistant Gram-negative bacteria. However, due to the emergence of the plasmid-mediated MCR-1 resistance gene, an increasing number of strains have developed resistance to polymyxin E. Additionally, the prevalence of *Escherichia coli* in chickens has imposed a heavy burden on the global food and poultry farming industries. This study aimed to investigate the in vitro and in vivo synergistic effects and mechanisms of quercetin (QUE) combined with polymyxin E against MCR-1-positive *E. coli* JD37 from chickens. The in vitro and in vivo synergistic effects of QUE and polymyxin E were confirmed through in vitro drug sensitivity tests, growth curves, time-kill curves, agar diffusion tests, and in vivo chick infection experiments. The results showed that QUE restored the sensitivity of polymyxin E to *E. coli* JD37 and restored the sensitivity of polymyxin E to *E. coli* JD37 mainly by increasing the permeability of the cell membrane, elevating the fluidity of the cell membrane, increasing the membrane potential, and reducing the expression of the AcrAB-TolC efflux pump and Lipopolysaccharide (LPS) regulatory-related genes. Molecular docking results revealed the key binding sites of QUE to MCR-1.

## 1. Introduction

Colibacillosis in chickens is one of the primary causes of morbidity and mortality in poultry, leading to various diseases such as yolk sac infection, respiratory infection, and septicemia, and causing significant economic losses to the global livestock industry [1]. However, with the overuse of antibiotics, the issues of multidrug resistance (MDR) and pandrug resistance (PDR) in *Escherichia coli* from chicken have become increasingly severe [2]. Polymyxin E, also known as colistin, is categorized into polymyxin A, B, C, and D based on its structure and origin. As a cyclic peptide antibiotic, it serves as the “last line of defense” against MDR and PDR Gram-negative bacterial infections. According to statistics, polymyxin E accounts for 30% of the usage in the treatment of intestinal infections across all species in European countries. In 2015, Shen Jianzhong and colleagues first discovered the polymyxin E resistance gene MCR-1 and demonstrated that MCR-1 could modify lipid A through phosphoethanolamine transferase, thereby reducing the net negative charge and mediating *E. coli*’s resistance to polymyxin E [3]. Additionally, its transmissibility between animals and humans poses a serious threat and challenge to the treatment of multidrug-resistant and extensively drug-resistant infections with polymyxin E [4]. At the same time, the neurotoxicity and nephrotoxicity of colistin caused by unreasonable dosage have also restricted its use [5]. However, the research by Reza Rajabalizadeh et al. [6] has demonstrated that the natural active component crocin can alleviate the renal damage in mice caused by the unreasonable dosage of colistin. Furthermore, research in 2024 revealed that the PhoPQ/pmrA/pmrB system could also regulate resistance through lipid A modification [7]. Therefore, addressing the issue of polymyxin E resistance has become an urgent challenge.

Currently, strategies to mitigate bacterial drug resistance primarily include the development of new antibiotics [8], combination therapy [9], phage therapy [10], and antimicrobial peptides [11]. However, the development of new antibiotics is a lengthy, challenging, and costly process; phage therapy presents a narrow host range and necessitates frequent administration [12]; and the extraction of antimicrobial peptides is complex, resulting in a relatively low utilization rate [13]. Among these approaches, antibiotic combination therapy stands out as a viable strategy to alleviate drug resistance at present. However, the irrational use of dosages, while effective in the short term, may lead to the enrichment of multiple drug resistance genes in the long term, resulting in greater harm to the liver and kidneys [14]. Yabes et al. found that the combination of aminoglycosides and vancomycin increases the incidence of acute kidney injury [15]. The combination of active ingredients from traditional Chinese medicine and antibiotics is currently a prominent topic in addressing existing challenges [16]. Active ingredients from traditional Chinese medicine exhibit multiple targets [17]. They not only enhance the antibacterial efficacy of antibiotics but also mitigate the toxic and side effects associated with antibiotic use, reverse drug resistance, and bolster the host’s immune defense capabilities [18]. Xiao-Die Cui et al. demonstrated that the combination of baicalin, EDTA, and polymyxin E enhances synergistic antibacterial activity against colistin-resistant Salmonella [19]. Additionally, Zhou [20] identified osthole as a potential synergist of polymyxin E, capable of restoring the sensitivity of polymyxin E to MCR-1-positive *E. coli* An increasing number of natural products derived from traditional Chinese medicine, including Cajanin, resveratrol, fisetin, pterostilbene, and Tetrandrine [21,22,23,24], have demonstrated inhibitory activity against MCR-1-positive *E. coli*.

Quercetin (QUE) is a flavonoid present in various fruits and vegetables. It exhibits a range of beneficial properties, including antioxidant [25], antibacterial [26], anti-inflammatory [27], and anti-cancer [28] effects. As a natural active ingredient with low toxicity, Patrice Cunningham et al. conducted a toxicity study on quercetin. Three different doses of quercetin (62, 125, and 250 mg/kg) were administered to mice for 98 days. The results showed that quercetin had no significant impact on the body composition, organ function, behavior, or metabolism of the mice [29]. Research conducted by Joycy F. S. Dos Santos et al. has demonstrated that QUE can enhance the efficacy of antibiotics by inhibiting the efflux pump of Staphylococcus aureus [30]. Elif Odabaş Köse et al. demonstrated that QUE can enhance the bactericidal effects of colistin and amikacin against drug-resistant Acinetobacter baumannii [31]. Similarly, Yishuai Lin et al. showed that QUE can restore the sensitivity of drug-resistant *E. coli* to colistin in both in vitro and in vivo settings [32]. However, there is currently a lack of clinical trial data to support the combined use of these medications.

This study investigated the synergistic effect and mechanism of QUE combined with polymyxin E against chicken-derived MCR-1-positive *E. coli* JD37 to assess its therapeutic potential. QUE restored bacterial susceptibility to polymyxin E by enhancing membrane permeability and fluidity, achieved through modulation of Lipopolysaccharide (LPS) content, reduction in phospholipase A2 activity, alteration of membrane potential, and inhibition of the AcrAB-TolC efflux pump. Molecular docking revealed that QUE exhibits a strong binding affinity with MCR-1. The synergistic efficacy was confirmed both in vitro and in a chick infection model. The toxicity and side effects of polymyxin E caused by dosage were alleviated through combined use with quercetin. These findings potentially discuss the underlying mechanism and support the further development of this combination therapy to combat polymyxin E resistance.

## 2. Materials and Methods

### 2.1. Strains and Animals

**Figure 1:**
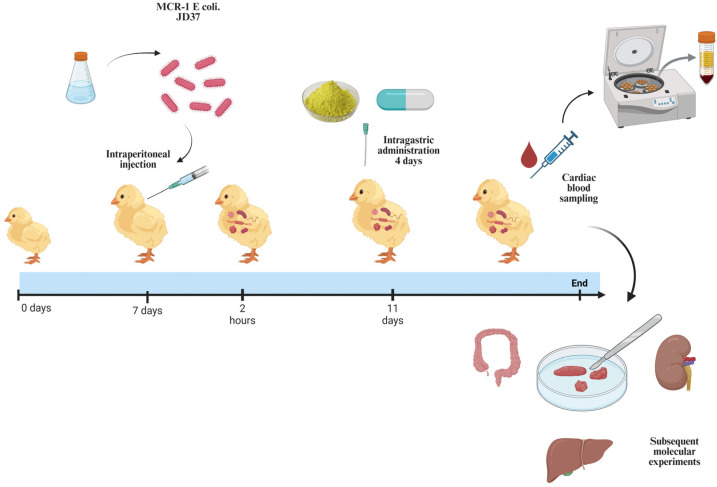
Experimental design of animals.

**Figure 2:**
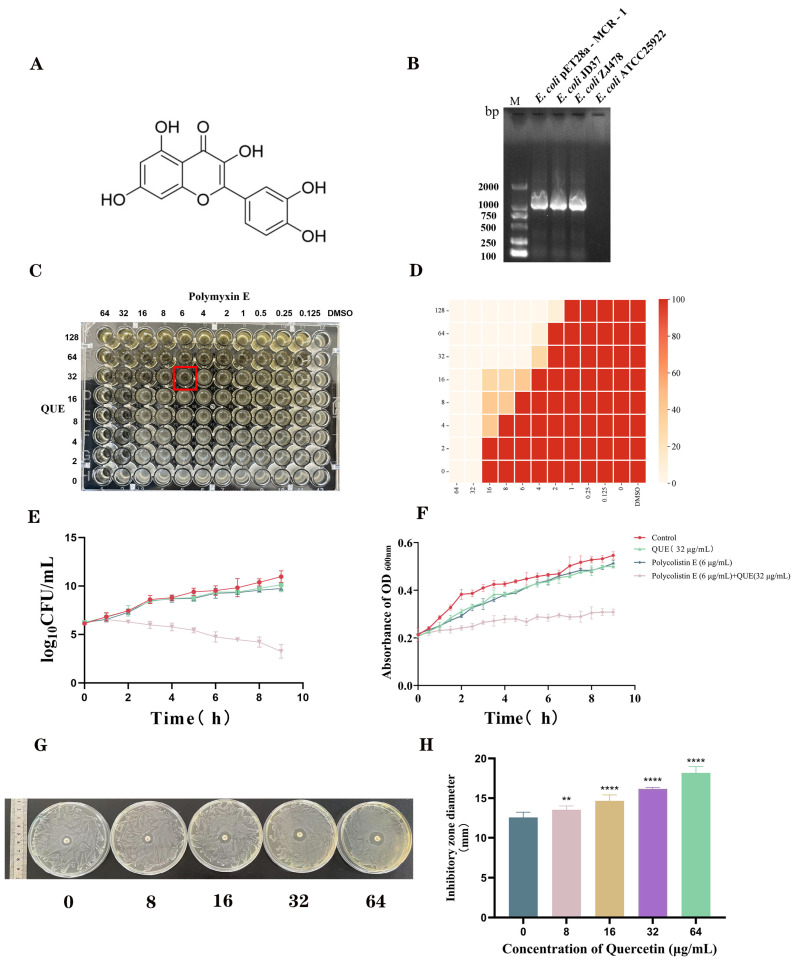
Chemical structure of QUE (**A**). PCR identification of the *E. coli* JD37 isolate. M: 2000 bp marker (**B**). Fractional inhibitory concentration (FIC) index determination of QUE and polymyxin E using checkerboard assay (**C**,**D**). Time-kill curves of combination therapy (**E**). Growth curves under combination treatment (**F**). Measurement of inhibition zone diameters post-combination therapy (**G**,**H**). The data are the mean ± S.D. of at least three independent experiments. ** represents *p* < 0.01, **** represents *p* < 0.0001.

*E. coli* ATCC25922 was purchased from the American Type Culture Collection (ATCC). *E. coli* pET-28a-mcr-1 was previously constructed in the laboratory. *E. coli* JD37 was clinically isolated from a farm in Harbin. *E. coli* ZJ478 was donated by Professor Wang Yang from China Agricultural University. One-day-old male Hy-Line Brown chicks were purchased from Harbin Guangda Chicken Farm. The animal experiment design is shown in Figure 1, All strains were identified by PCR (Figure 2B).

### 2.2. Chemicals and Reagents

Polymyxin E (CAS No.1264-72-8) was purchased from Yeasen Biotechnology (Shanghai) Co., Ltd. (Shanghai, China), QUE (CAS No.117-39-5) was purchased from Shanghai Aladdin Biochemical Technology Co., Ltd. (Shanghai, China), dimethyl sulfoxide (DMSO) was purchased from Beijing Bio-Tech Solutions Co., Ltd. (Beijing, China), the antibiotic susceptibility disks of polymyxin E were purchased from liofilchem (Rome, Italy), the LPS ELISA kit (No.AD11746Hu) was purchased from Andy Gene Biotechnology Co., Ltd. (Shanghai, China), SYTO-9 (Cas No: S33846) was purchased from White Shark Biotechnology Co., Ltd. (Hefei, China), PI (propidium iodide CAS No: H292833) was purchased from White Shark Biotechnology Co., Ltd., Laurdan (CAS No.74515-25-6) was purchased from MedChemExpress (Monmouth Junction, NJ, USA), the phospholipase A2 activity assay kit (No.AD8201Hu) was purchased from Andy Gene Biotechnology Co., Ltd., DiOC2-(3) (CAS: 1895296-01-1) was purchased from Shanghai Aladdin Biochemical Technology Co., Ltd., and the IL-1β, IL-6, and TNF-α ELISA kits were purchased from Beijing Chenglin Biotechnology (Beijing, China).

### 2.3. Combined Drug Sensitivity Test

The synergistic effect between QUE and polymyxin E against chicken-derived MCR-1-positive *E. coli* was assessed using the checkerboard microdilution method [30]. Bacterial suspensions were adjusted to 1 × 10^5^ CFU/mL in LB broth. In a 96-well plate, polymyxin E (0–64 μg/mL) and QUE (0–128 μg/mL) were serially diluted along the X and Y axes, respectively. Each well was inoculated with 5 μL of bacterial suspension. After 16 h of incubation at 37 °C, the MIC was recorded as the lowest concentration showing no visible growth. The fractional inhibitory concentration (FIC) index was calculated as described [31].

### 2.4. Time-Kill Curves

A bacterial suspension (1 × 10^5^ CFU/mL) was treated with polymyxin E (6 μg/mL), QUE (32 μg/mL), and their combination and incubated at 37 °C. At hourly intervals, 100 μL aliquots were plated onto LB agar containing polymyxin E. After 16 h of incubation, bacterial colonies were enumerated to generate time-kill curves.

### 2.5. Growth Curves

The strain under investigation was cultured in LB broth at 37 °C in a shaker until the optical density (OD) reached 0.3. Subsequently, it was exposed to polymyxin E at a final concentration of 6 μg/mL, QUE at a final concentration of 32 μg/mL, and a combination of polymyxin E (6 μg/mL) and QUE (32 μg/mL). The OD was measured at a wavelength of 600 nm every half hour to assess the effects on bacterial growth.

### 2.6. Agar Diffusion Test

QUE was added to LB medium to prepare solutions at final concentrations of 0, 8, 16, 32, and 64 μg/mL. Subsequently, LB agar Petri dishes were prepared. A bacterial suspension with a concentration of 1 × 10^5^ CFU/mL was uniformly spread across the plates. An antibiotic susceptibility disk containing 10 μg of polymyxin E was positioned at the center of the plate surface. The plates were incubated at 37 °C for 24 h. The diameter of the inhibition zone was measured using a vernier caliper and recorded for comparison.

### 2.7. Membrane Permeability Detection

#### 2.7.1. The Determination of LPS-Related Regulatory Genes and LPS Content Involved Co-Incubating a Bacterial Suspension

Bacterial suspensions (1 × 10^8^ CFU/mL) were treated for 2 h with QUE (32 μg/mL), polymyxin E (6 μg/mL), and their combination. After incubation, cells were collected by centrifugation (8000 rpm, 5 min) and resuspended in PBS (pH 7.2–7.4). Total RNA was extracted using TRIzol (Beijing Bioray Biotechnology Co., Ltd., Beijing, China), and cDNA was synthesized with the PrimeScript™ RT reagent Kit (Beijing Bioray Biotechnology Co., Ltd., Beijing, China). Quantitative PCR was performed using TB Green^®^ Premix Ex Taq™ II, with 16S rRNA as the internal reference, and efflux pump gene expression was analyzed via the 2^(–ΔΔCq)^ method. Primer sequences are shown in Table 1. Subsequently, LPS content was quantified using an ELISA kit (Beijing Chenglin Biotechnology Co., Ltd., Beijing, China), with a standard curve for analysis and an untreated group as a negative control.

#### 2.7.2. Cell Membrane Staining

Bacterial suspensions were adjusted to 1 × 10^8^ CFU/mL and treated with QUE (32 μg/mL), polymyxin E (6 μg/mL), and their combination for 2 h. Cells were then pelleted by centrifugation (8000 rpm, 5 min), washed, and resuspended in 0.85% NaCl. Subsequently, 3 μL of a fluorescent dye mixture (SYTO-9 and PI) was added per mL of bacterial suspension, followed by incubation in the dark for 15 min. A 5 μL aliquot of the stained suspension was placed on a glass slide for observation under a fluorescence microscope (Leica DM-i8 from Germany).

#### 2.7.3. Scanning Electron Microscopy Analysis

Bacterial suspensions were adjusted to a concentration of 1 × 10^8^ CFU/mL and treated with polymyxin E (6 μg/mL), QUE (32 μg/mL), and their combination for 4 h. The samples were then fixed with 5% glutaraldehyde and prepared for observation by scanning electron microscopy (TM4000, Hitachi, Tokyo, Japan).

### 2.8. Measurement of Membrane Fluidity

Bacterial suspensions (1 × 10^8^ CFU/mL) were treated with QUE (32 μg/mL), polymyxin E (6 μg/mL), and their combination for 2 h. After centrifugation (8000 rpm, 5 min), the pellet was resuspended in PBS and stained with the Laurdan fluorescent probe for 30 min. Membrane fluidity was then assessed by measuring fluorescence polarization using a fluorescence spectrophotometer (Hitachi F-7100, Japan). The Generalized Polarization (GP) value was calculated using the formula: GP = (I_440_ − I_490_)/(I_440_ + I_490_), where I_440_ and I_490_ represent the emission intensities at 440 nm and 490 nm, respectively, upon excitation at 360 nm.

### 2.9. Determination of Phospholipase A2 Activity

Phospholipase A2 activity was measured in bacterial suspensions (1 × 10^8^ CFU/mL) after 2 h of treatment with QUE (32 μg/mL), polymyxin E (6 μg/mL), and their combination. Following incubation, cells were collected by centrifugation (8000 rpm, 5 min), resuspended in PBS (pH 7.2–7.4), and analyzed using a commercial assay kit.

### 2.10. Measurement of Membrane Potential

Bacterial suspensions (1 × 10^8^ CFU/mL) were treated with QUE (32 μg/mL), polymyxin E (6 μg/mL), and their combination for 2 h. Cells were harvested by centrifugation (8000 rpm, 5 min), resuspended in PBS, and stained with 5 μM DiOC_2_-(3) for 20 min at 37 °C in the dark. Membrane potential was assessed by fluorescence microscopy (Leica DM-i8 from Germany) after slide preparation. The shift from green to red fluorescence indicates membrane hyperpolarization. Fluorescence intensity was quantified using ImageJ 1.54g software.

### 2.11. Detection of AcrAB-TolC Efflux Pump

Bacterial suspensions (1 × 10^8^ CFU/mL) were treated with QUE (32 μg/mL), polymyxin E (6 μg/mL), and their combination for 6 h. Total RNA was extracted using TRIzol and reverse-transcribed into cDNA with the PrimeScript™ RT reagent Kit. Quantitative PCR was performed using TB Green^®^ Premix Ex Taq™ II, with 16S rRNA as the endogenous control. The relative expression of efflux pump-related genes was calculated using the 2^(–ΔΔCq)^ method. Primer sequences are shown in Table 1.

### 2.12. Molecular Docking

The crystal structure of the MCR-1 protein (RCSB PDB_ Homepage.html, PDB ID: 5GRR) was retrieved from the Protein Data Bank. Using PyMOL 2.4.0, water molecules and hetero ligands were removed from the protein structure, which was then prepared for docking using AutoDockTools 4.2.6. Hydrogen atoms were added, Gasteiger charges were assigned, and the file was converted to PDBQT format. Molecular docking of QUE to MCR-1 was performed using AutoDock Vina 4.2.6.

### 2.13. Animal Experiments

After 7 days of acclimation, one-day-old chicks were randomly allocated into six groups (n = 10 per group): a control group, an infected group, a QUE group (75 mg/kg), a polymyxin E group (50 mg/kg), one group treated with a combination of quercetin (75 mg/kg) and polymyxin E (50 mg/kg), and another group treated with a combination of quercetin (150 mg/kg) and polymyxin E (50 mg/kg)., Except for the control group injected with saline, all chicks were intraperitoneally challenged with 0.2 mL of *E. coli* JD37 (2 × 10^9^ CFU/mL) (Figure 1). Treatments were administered orally once daily, starting 2 h post-infection, for 4 days. Survival was monitored every 12 h. On day 4, surviving chicks were euthanized. Bacterial loads in the liver, kidney, and cecum were quantified by plating homogenates on polymyxin E-containing LB agar. Cecal levels of IL-1β, IL-6, and TNF-α were measured by ELISA. Tissues (liver, cecum, kidney) were fixed in formalin for H&E staining and histopathological examination.

The experimental program was conducted in accordance with the regulations of the Ethics Committee for Experimental Animals of Northeast Agricultural University and the requirements of the Guidelines for the Welfare and Ethical Review of Laboratory Animals (GB/T 35892-2018 [25], Harbin National Standard of the People’s Republic of China). The animals were housed in an environment with a temperature of 25 ± 2 °C, humidity of 55 ± 10%, absence of specific pathogens, light/darkness for 12 h each, and were provided with ad libitum access to food and water.

### 2.14. Statistical Analysis

The experimental data were analyzed using GraphPad Prism 10.12, with each treatment group replicated three times in parallel. One-way ANOVA was employed to determine significance, and * represents *p* < 0.05, ** represents *p* < 0.01, *** represents *p* < 0.001, and **** represents *p* < 0.0001.

## 3. Results

### 3.1. QUE Restored the Sensitivity of E. coli JD37 to Polymyxin E

To evaluate the synergy between QUE (Figure 2A) and polymyxin E, we used a chicken-derived MCR-1-positive *E. coli* JD37 (Figure 2B). The initial MICs were 64 μg/mL for polymyxin E and 128 μg/mL for QUE. Checkerboard assays revealed strong synergy, with an FIC index of 0.34375 (Figure 2C,D). This synergy was further supported by time-kill curves, which showed a significant bactericidal effect within 9 h only with the combination of 6 μg/mL polymyxin E and 32 μg/mL QUE (Figure 2E). Growth curve analysis indicated that the combination, but not the individual agents, effectively suppressed bacterial growth over time (Figure 2F). Additionally, in agar diffusion assays, the inhibition zone diameter around a polymyxin E disk (10 μg/mL) increased significantly from 12.25 ± 0.25 mm to 18.17 ± 0.33 mm as the QUE concentration increased to 64 μg/mL (*p* < 0.001; Figure 2G,H).

### 3.2. QUE Enhances the Permeability of the Bacterial Cell Membrane

**Figure 3:**
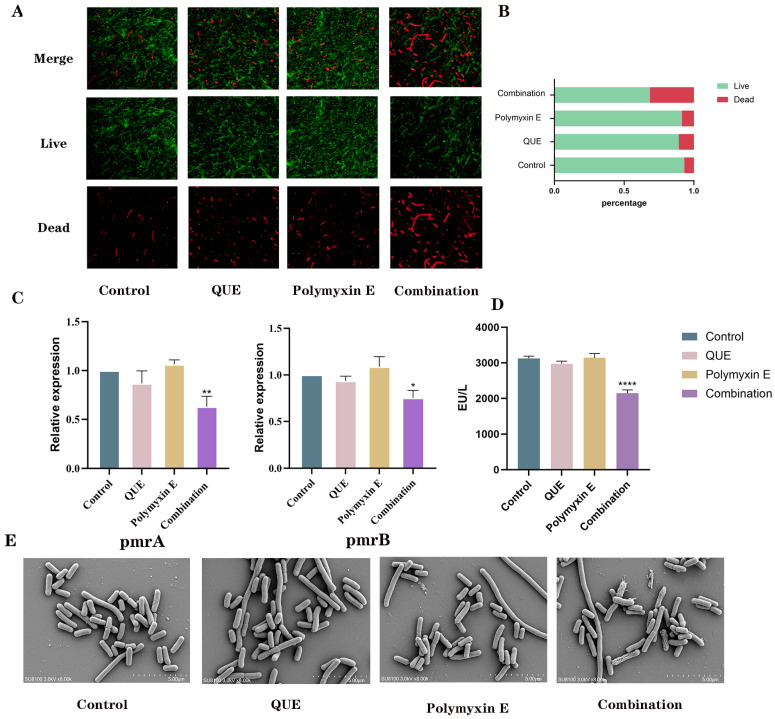
SYTO-9/PI staining results (**A**). Quantitative analysis of SYTO-9/PI staining (**B**). Relative mRNA expression levels of pmrA and pmrB as determined by RT-qPCR (**C**). LPS content measured using an LPS detection kit (**D**). Scanning electron microscopy analysis of bacterial cell membranes (**E**). The data are the mean ± S.D. of at least three independent experiments. * represents *p* < 0.05, ** represents *p* < 0.01, **** represents *p* < 0.0001.

As shown in (Figure 3A,B), the combination of QUE and polymyxin E significantly compromised bacterial membrane integrity, as indicated by a marked increase in red fluorescence and a decrease in green fluorescence compared to the minimal effects of either agent alone. This increase in membrane permeability was accompanied by significant morphological changes. Scanning electron microscopy revealed severe membrane shrinkage, cell lysis, and loss of normal rod shape in the combination group, in contrast to the intact, smooth surfaces of bacteria treated with single agents (Figure 3E). At the molecular level, the combination therapy significantly downregulated the expression of the regulatory genes pmrA (*p* < 0.05) and pmrB (*p* < 0.01), an effect not observed with individual treatments (Figure 3C). Consistent with these findings, LPS content was most substantially reduced in the combination group (*p* < 0.001; Figure 3D), suggesting that the synergy arises from the coordinated disruption of the outer membrane and its regulatory pathways.

### 3.3. QUE Changes the Fluidity of the Bacterial Cell Membrane

**Figure 4:**
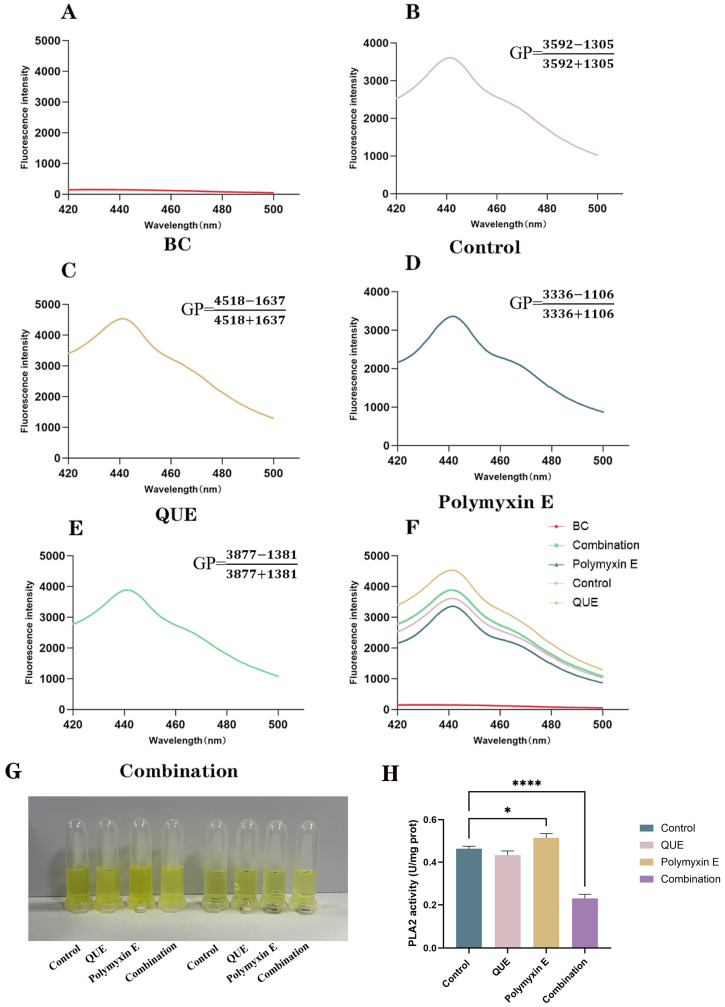
Fluorescence polarization GP values for the different experimental groups (**A**–**F**). Enzyme activity was assessed using a phospholipase A2 assay kit (**G**,**H**). The data are the mean ± S.D. of at least three independent experiments. * represents *p* < 0.05, **** represents *p* < 0.0001.

The impact on membrane fluidity was assessed by measuring the generalized polarization (GP) value. While treatment with QUE alone had little effect, polymyxin E significantly increased the GP value, indicating reduced membrane fluidity. However, co-administration with QUE lowered the GP value compared to polymyxin E alone, demonstrating that QUE enhances membrane fluidity (Figure 4A–F). We further investigated PLA2 activity, as it influences lipid composition and fluidity. The combination treatment significantly suppressed PLA2 activity (*p* < 0.001), more so than either agent alone (Figure 4G,H). This suppression is predicted to increase the proportion of unsaturated phospholipids, thereby enhancing membrane fluidity and permeability, which likely contributes to the restored sensitivity to polymyxin E.

### 3.4. QUE Changed the Membrane Potential of Bacteria

**Figure 5:**
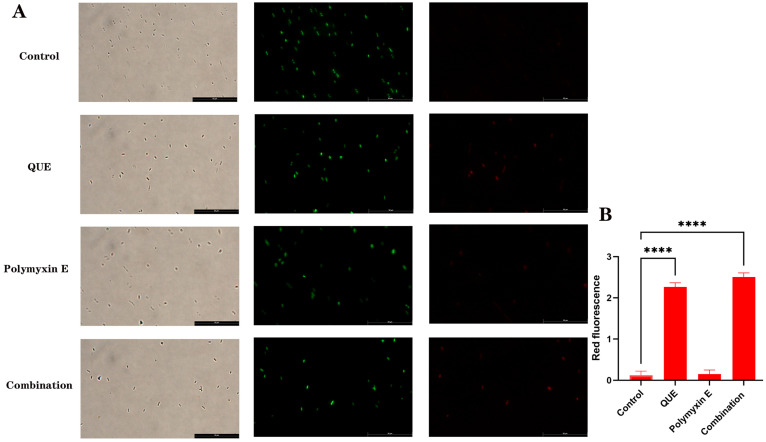
Analysis of DiOC2 (3) fluorescence intensity in different groups (**A**). Red fluorescence quantification results (**B**). The data are the mean ± S.D. of at least three independent experiments, **** represents *p* < 0.0001. Scale bar: 50 μm (**A**).

The results are presented in Figure 5. Following treatment with QUE alone, the fluorescent dye demonstrated self-aggregation, accompanied by an increase in membrane potential. This effect was notably more pronounced in the group receiving combined drug treatment. Our findings suggest that the concurrent administration of QUE and polymyxin E significantly modifies the membrane potential of avian *E. coli* JD37.

### 3.5. QUE Suppresses the Expression of Genes Associated with the AcrAB-TolC Efflux Pump System in Bacteria

**Figure 6:**
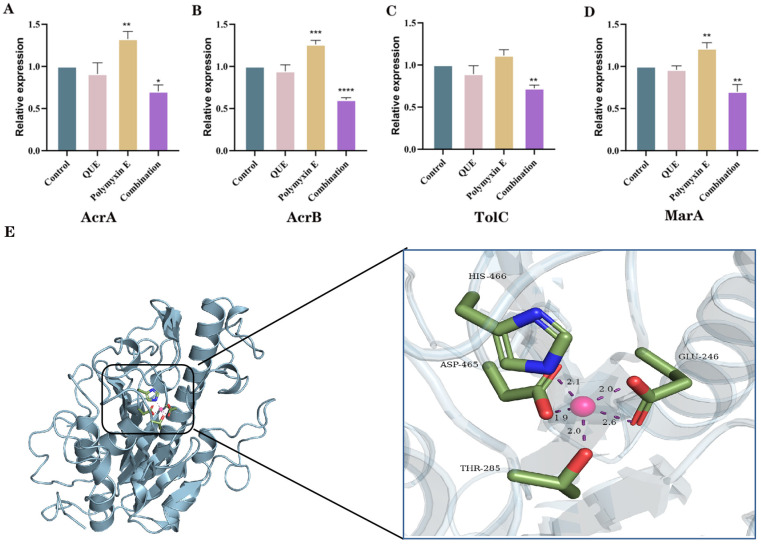
RT-qPCR determination of the relative transcriptional levels of AcrA, AcrB, TolC, and MarA in the AcrAB-TolC efflux pump system (**A**–**D**). Molecular docking visualization of QUE with the MCR-1 resistance gene (**E**). The data are the mean ± S.D. of at least three independent experiments, * represents *p* < 0.05, ** represents *p* < 0.01, *** represents *p* < 0.001, and **** represents *p* < 0.0001.

The results are presented in Figure 6. Our findings indicate that QUE, when administered alone, slightly inhibited the expressions of AcrA, AcrB, TolC, and MarA (*p* > 0.05). In contrast, the administration of polymyxin E alone resulted in an increase in the expression of AcrA (*p* < 0.05) and MarA, with varying degrees of significance. Notably, when QUE was combined with polymyxin E, there was a significant decrease in the expressions of AcrA, AcrB, TolC, and MarA (*p* < 0.05). These results demonstrate that QUE can restore the sensitivity of MCR-1-positive avian *E. coli* to polymyxin E by inhibiting the expression of efflux pump genes.

### 3.6. The Binding Site of QUE on MCR-1

Molecular docking results are illustrated in Figure 6E. QUE primarily interacts with residues including HIS-466, ASP-465, THR-285, and GLU-246 via hydrogen bonds. The molecular docking analysis reveals that the binding energy between QUE and the MCR-1 resistance gene is −5.6 kcal/mol, which is below the threshold of −5 kcal/mol, and the hydrogen bond distances are consistently less than 3 Å. These findings suggest that QUE exhibits a strong binding affinity for MCR-1 [33].

### 3.7. QUE in Combination with Polymyxin E Demonstrates Protective Efficacy in Chickens Infected with E. coli JD37

**Figure 7:**
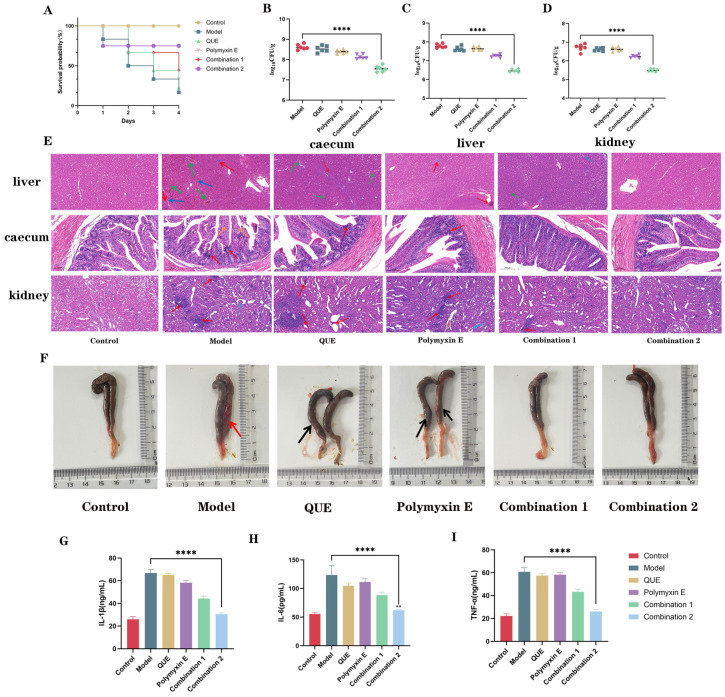
Survival rate of chicks following challenge (**A**). Bacterial load in the cecum, liver, and kidney (**B**–**D**). H&E-stained pathological sections of the liver, cecum, and kidney (**E**), Red arrows: Inflammation; green arrows: Appearance of congestion; dark blue arrows: Vacuoles appear in liver cells; oranges: Decrease in goblet cells; light blues: Tuberculous nodules.Gross postmortem observations of the cecum; black arrows indicate swelling, and red arrows indicate hemorrhagic foci (**F**), Red arrow: Bleeding point; Black arrows: Enlargement occurs Levels of IL-1β, IL-6, and TNF-α as determined by detection assays (**G**–**I**). The data are the mean ± S.D. of at least three independent experiments. **** represents *p* < 0.0001. Scale bar: 100 μm (**E**).

The efficacy of the combination therapy was further validated in a chick infection model. While the model group suffered an 80% mortality rate, the combination of QUE and polymyxin E significantly improved survival, with the higher-dose QUE combination 2 achieving over 65% survival, compared to below 25% for polymyxin E alone (Figure 7A). Combination 2 also markedly reduced bacterial loads in the cecum, liver, and kidneys (Figure 7B–D). Histopathological analysis revealed that the combination therapy restored intestinal architecture, increased goblet cells, and attenuated liver and kidney damage observed in the model group (Figure 7E). Macroscopic examination confirmed improved cecal morphology in the combination groups (Figure 7F). Furthermore, Group 2 significantly lowered cecal levels of the pro-inflammatory cytokines IL-1β, IL-6, and TNF-α (Figure 7G–I), demonstrating a potent anti-inflammatory effect.

## 4. Discussion

The increasing prevalence of the MCR-1 gene in Gram-negative bacteria has severely limited therapeutic options, prompting the search for strategies to restore the efficacy of last-line antibiotics like polymyxin E [34]. Here, we demonstrate that the natural product QUE effectively resensitizes chicken-derived *E. coli* to polymyxin E, reducing its MIC by nearly 11-fold from 64 μg/mL to 6 μg/mL. Moreover, it has shown significant efficacy in our in vivo experiments, providing substantial guidance for the veterinary clinical treatment of chicken-derived drug-resistant *E. coli* infections.

QUE, a naturally occurring flavonoid, exhibits weak antibacterial activity against *E. coli* [35]. Previous studies identified catechol-type flavonoids as potential synergists for colistin and proposed targeting bacterial iron homeostasis as an innovative strategy for developing novel antibacterial adjuvants [36]. This study explored the synergistic effect between QUE and polymyxin E, suggesting that natural flavonoids may enhance the efficacy of antibiotics against drug-resistant bacteria through a common mechanism. These findings provide new insights into combination therapies for multidrug-resistant bacterial infections.

The efflux pump is one of the core mechanisms of bacterial drug resistance, and overcoming the function of the efflux pump is also a way to alleviate drug resistance [37]. Molecular docking has revealed that QUE has the potential to inhibit the efflux pump activity of carbapenem-resistant Gram-negative bacteria [38]. This prompts us to consider whether QUE can restore the sensitivity of polymyxin E to *E. coli* by inhibiting the efflux pump. The most representative efflux pump in *E. coli* is AcrAB-TolC [39], which consists of three parts: AcrA (periplasmic adaptor protein), AcrB (inner membrane transporter protein), and TolC (outer membrane channel protein). These three work in concert to form a transmembrane channel that expels drugs from within the bacteria. This can reduce the concentrations of antibiotics such as tetracyclines [40] and carbapenems [41]. MarA is the main regulatory factor of the AcrAB-TolC efflux pump in *E. coli* [42]. Therefore, in this experiment, the genes related to the AcrAB-TolC efflux pump of *E. coli* were detected.

Meanwhile, the functioning of efflux pumps also relies on the supply of ATP [43]. The bacterial membrane potential is a key factor driving ATP synthesis [44], and the membrane potential also has a certain impact on bacterial drug resistance. For example, the membrane potential of *E. coli* is associated with resistance to dihydrostreptomycin [45]. The alteration of cell membrane potential is also closely related to bacterial metabolism. Zhang et al.’s [46] research indicates that when the membrane potential of bacterial cells decreases, fatty acids enter the bacteria, thereby providing nutrients for the bacteria, influencing their metabolism, and enhancing their survival rate. Additionally, one of the resistance mechanisms of MCR-1 is to rely on PEA to modify lipid A and reduce the electronegativity of the outer cell membrane [3]. Bacterial cell membrane potential, as one of the core driving forces of cellular physiological activities, plays a key role in the formation of antibiotic resistance. Recent studies have found that many antibiotic resistances are related to membrane potential, such as aminoglycosides [47] and tetracycline [48]. Altering membrane potential provides a new approach to intervening in the antibiotic resistance of *E. coli*.

In addition, LPS is an important barrier and macromolecule in the outer membrane of bacteria. Changes in its content affect the permeability of the cell membrane. The classical mechanism of action of polymyxin E is to target the lipopolysaccharide in the outer membrane of Gram-negative bacteria. QUE can alleviate LPS-induced liver inflammation [49]. When the synthesis of lipopolysaccharide is reduced, the permeability of the bacterial cell membrane increases. When the permeability increases, it becomes easier for antibiotics to enter the bacteria [50]. The LPS modification regulated by the PmrA/PmrB system is an important mechanism for bacteria to resist “last line of defense” antibiotics such as polymyxin E, and is also known as the “LPS regulator” [51]. Understanding the relationship between the PmrA/PmrB system and LPS regulation not only helps to reveal the bacterial resistance mechanism but also provides an important basis for the development of new antibacterial strategies. The permeability of the cell membrane is also closely related to the efflux pump, the fluidity of the cell membrane, and the membrane potential. The efflux pump and the membrane potential are the energy sources of the cell membrane. By changing the membrane potential, the energy metabolism of the efflux pump is affected, thereby altering the permeability of the bacterial cell membrane [52]. The fluidity provides structural interaction for the efflux pump [53]. In general, there is a close and complex relationship between efflux pumps, membrane fluidity, membrane potential, membrane permeability, and drug resistance, and further exploration is needed.

Meanwhile, multidrug-resistant bacteria (MDR) can also enhance their drug resistance by altering the fluidity of the cell membrane [54]. When the fluidity of the cell membrane increases, the permeability of antibiotics also increases, greatly increasing the chance of entering the cell. Haihua Yuan et al. [34] found that QUE could disrupt the synthesis of bacterial fatty acids by inhibiting β-ketoacyl-acyl carrier protein reductase (FabG), a potential target in the bacterial fatty acid biosynthesis pathway, thereby changing the fluidity of the cell membrane and affecting the bacteria’s own metabolism. Phospholipase A2 (PLA2) is a key regulatory enzyme for the fluidity of the bacterial cell membrane. It hydrolyzes the ester bond at the sn-2 position of membrane phospholipids to generate lysophospholipids and free fatty acids, directly regulating the physicochemical properties and fluidity of the cell membrane [55]. In the acute kidney injury model of rats, it was found that the addition of PLA2 inhibitors significantly reduced tissue damage and inflammation [56]. Meanwhile, PLA2 also plays a crucial regulatory role in cell fluidity [57]. The fluidity of the cell membrane is related to the ratio of saturated to unsaturated fatty acids [58], temperature [59], and protein interactions [60]. In addition, changes in fluidity are associated with multiple drug resistance mechanisms. For instance, Bacillus atrophaeus can enhance its tolerance to antibiotics by reducing membrane fluidity [61]. Therefore, research on the regulatory mechanisms of membrane fluidity may provide new targets for reversing bacterial drug resistance.

Molecular docking technology, by simulating the interaction between drug molecules and target proteins, can precisely analyze the changes in the binding mode of drugs and target proteins, and has become a key method for studying the mechanism of drug resistance and discovering new antibiotic potentiators [62]. The natural active ingredient euphorbol D disrupts Mycoplasma gallisepticum infection through interactions with amino acid residues Cys158, His157, and Asp211, and shows a strong binding affinity for TatD nuclease [63]. In the experiment of screening pyrazolinone compounds as candidate drugs for MCR-1 inhibitors through computer simulation and in vitro studies, it was found that GLU-246, THR-285, HIS-395, HIS-466, and HIS-478 are the key active sites for MCR-1 to exhibit drug resistance [64]. Molecular docking provides new methods and strategies for addressing drug resistance.

However, this study has a potential limitation. In the original experimental design, there was no separate in vivo toxicity control group, which led to the inability to completely rule out the damage caused by the drug itself. Therefore, in future research, we need to further explore this issue.

## 5. Conclusions

Our research demonstrates that the co-administration of QUE and polymyxin E effectively inhibits *E. coli* positive for MCR-1 derived from chickens in vitro. When the concentration of QUE reaches 32 μg/mL, the minimum inhibitory concentration (MIC) of polymyxin E against chicken-derived MCR-1-positive *E. coli* decreases from 64 μg/mL to 6 μg/mL, representing an almost 11-fold reduction. The combination of 50 mg/kg polymyxin E and 150 mg/kg QUE significantly inhibits the infection of chicken-derived MCR-1-positive *E. coli* in chicks and improves their survival rate. The mechanism by which polymyxin E sensitivity is restored involves enhancing cell membrane permeability, increasing membrane fluidity, and altering membrane potential, which collectively affect the energy metabolism of the efflux pump. Concurrently, this combination reduces the expression of genes associated with the efflux pump and LPS regulation, thereby enhancing the sensitivity of polymyxin E against chicken-derived *E coli.* Finally, molecular docking studies were conducted to predict the site of action for QUE, providing a reference for subsequent experiments. The molecular docking results revealed that residues HIS-466, ASP-465, THR-285, and GLU-246 are the key binding sites for quercetin with MCR-1.

## Figures and Tables

**Table 1:** Primer sequences for qRT-PCR.

Primer Name	Oligonucleotide (5′-3′)
acrA	F:CTTAGCCCTAACAGGATGTG R:TTGAAATTACGCTTCAGGAT
acrB	F:GAGAAGAGCACGCACCACTACAC R:GGCAGACGCACGAACAGATAGG
TolC	F:GGTACGTTGAACGAGCAGGATC R:CCATCAGCAATAGCATTCTGTTCC
PmrA	F:CCTTTTGCGCTGGAAGAGT R:TCTTTGGGCGTCAGAATCAAC
PmrB	F:CTGCAAGAAGATGACGGAGC R:CTGTGTAATGCGGCTGACCA
16sRNA	F:CTCTTGCCATCAGATGTGCC R:TTCTTCATACACGCGGCATG
MCR-1	F:ATGATGCAGCATACTTCTGTGTG R:TCAGCGGATGAATGCGGTGC

## Data Availability

All data in this article are presented in the article.

## Abbreviations

The following abbreviations are used in this manuscript:

QUE	Quercetin
E. coli	Escherichia coli
HIS	histidine
ASP	Linear dichroism
THR	Threonine
GLU	Glutamate
IL-6	interleukin-6
IL-1β	iinterleukin-1 beta
TNF-α	Tumor Necrosis Factor-alpha
LPS	Lipopolysaccharide
GP	generalized polarization
PDR	pandrug resistance
MDR	multidrug resistance
